# Peripheral Lung Squamous Carcinoma With ROS1 Rearrangement Sensitive to Crizotinib: A Case Report

**DOI:** 10.3389/fonc.2021.703318

**Published:** 2021-06-21

**Authors:** Guangdie Yang, Jie Wang, Yinan Yao, Jun Zhao, Zheyan Yu, Qiqi Gao, Jiani Ye, Wenjiang Ma

**Affiliations:** ^1^ Department of Respiratory Medicine, The First Affiliated Hospital, College of Medicine, Zhejiang University, Hangzhou, China; ^2^ Department of Respiratory Medicine, Zhejiang Medical & Health Group Hangzhou Hospital, Hangzhou, China; ^3^ Department of Respiratory and Critical Care Medicine, Shengzhou People’s Hospital, Shaoxing, China; ^4^ Department of Pathology, The First Affiliated Hospital, College of Medicine, Zhejiang University, Hangzhou, China

**Keywords:** lung squamous carcinoma, ROS1 rearrangement, crizotinib, atypical imaging manifestation, hypoalbuminemia

## Abstract

ROS1 rearrangements have been identified as driver mutations, accounting for 1–2% of lung adenocarcinoma, but are extremely rare in case of lung squamous cell carcinoma. In this work, we report a lung squamous cell carcinoma in a patient with peripheral lung cancer radiological manifestation, harboring ROS1 rearrangement, with high sensitivity to crizotinib. Our findings suggest that clinicians should pay more attention toward the occurrence of ROS1 rearrangements and the application of crizotinib for lung squamous cell carcinoma treatment.

## Introduction

In the last few decades, genetic testing and targeted therapy have resulted in survival benefits among patients with lung adenocarcinoma, although progress in the treatment of lung squamous cell carcinoma (SCC) remains stagnant. Recently, there has been a growing biological significance to identify the molecular characteristics of patients with lung SCC. ROS1 is a proto-oncogene and one among the sevenless subfamily of tyrosine kinase insulin receptor genes. ROS1 rearrangements are a known oncogenic driver in 1–2% of patients with lung adenocarcinoma, while it is widely believed that ROS1 in SCC is very rare (1). Crizotinib is a standard treatment for adenocarcinomas with ROS1 rearrangement (2), although we still have no data regarding the application of crizotinib in patients with lung SCC. Here, we report a rare case of a non-smoker female patient, diagnosed with peripheral lung SCC harboring ROS1 rearrangement, who was extremely sensitive to crizotinib.

## Case Description

**Figure 1:**
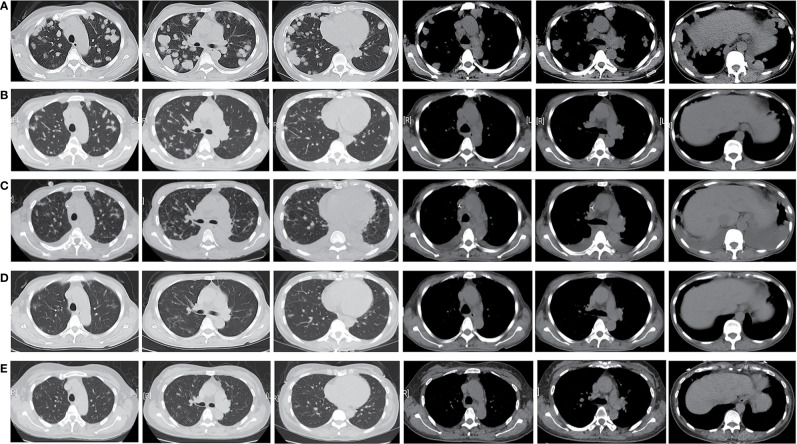
Timeline of the patient’s chest CT scan showed the obvious remission of the patient’ s diffused lesions in the bilateral lung and enlarged lymph nodes after crizotinib treatment: **(A)** 7 July, 2020 (baseline), **(B)** 24 August, 2020, **(C)** 22 September, 2020, **(D)** 14 December, 2020, **(E)** 25 April, 2021.

**Figure 2:**
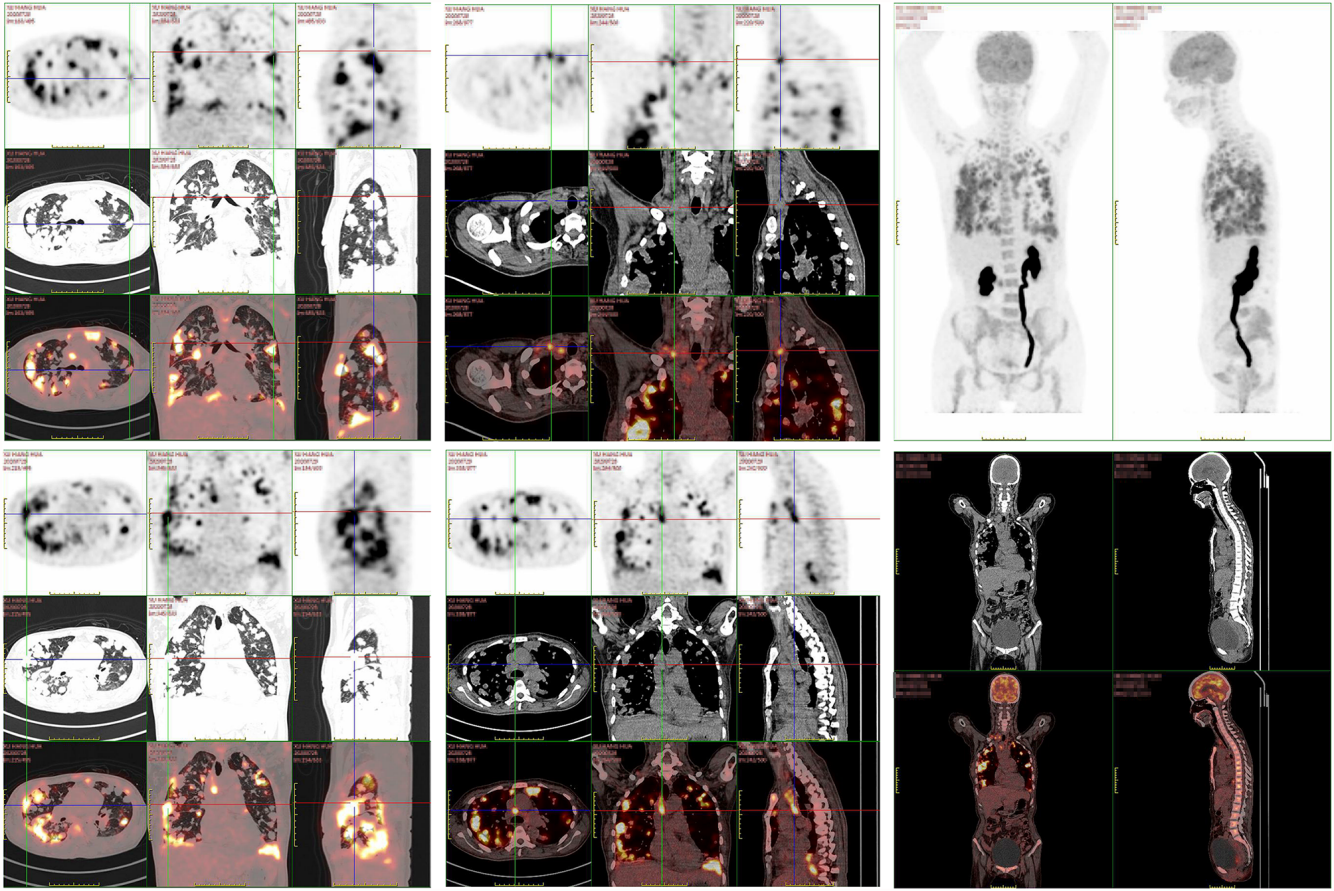
The patient’ s PET/CT scan showed diffused lesions with increased FDG uptake like chest CT located in the lung and LNs without distal metastasis.

**Figure 3:**
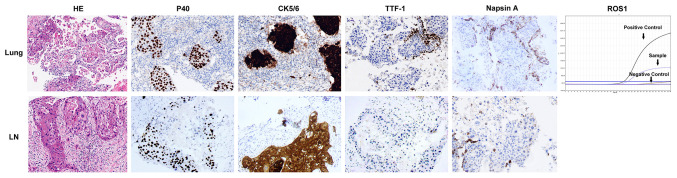
Pathological and molecular examinations of the case. HE staining showed the microscopic appearance of squamous cell carcinoma with nests of polygonal cells with pink cytoplasm and distinct cell borders (×200). Immunostaining of lung tissue and supraclavicular lymph node showed strongly positive for p40 and CK5/6, and negative for TTF-1 and Napsin A (×200). ARMS assay of lung tissue showed ROS1 rearrangement.

A 47-year old woman presented with repeated fever and fatigue in the past 3 months. She had a history of idiopathic thrombocytopenic purpura (ITP) and had undergone splenectomy 5 years ago with no evidence of recurrence. She was a non-smoker. Bilateral cervical lymph nodes (LNs) were palpable, and other physical examinations showed no abnormalities. Chest computed tomography (CT) scan showed diffuse round high-density lesions with small pleural effusion in the lungs bilaterally, accompanied by multiple enlarged LNs in the mediastinum and right supraclavicular area (**Figure 1A**). Ultrasound evaluation of the cervical LNs suggested bilateral supraclavicular lymphadenopathy (size in the right was 2.6 cm × 1. 6cm, size in the left was 2.2 cm × 1.1 cm). Abdominal CT, transvaginal ultrasound and cranial magnetic resonance (MR) imaging were normal. The results of representative serum tumor markers were as follows: CEA 7.6 ng/ml (normal < 5.0 ng/ml), CYFRA21 >100.0 ng/ml (normal < 3.3 ng/ml), NSE 17.6 ng/ml (normal < 30 ng/ml), SCCA >70.0 ng/ml (normal < 1.5 ng/ml). Positron emission tomography (PET)–CT showed no distal metastasis, which showed similar lesions with increased Fluorodeoxyglucose (FDG) uptake as chest CT in the lung and LNs (N2, N3) (**Figure 2**). Therefore, she underwent ultrasound-guided biopsy of the lung and right supraclavicular LNs. Tissue histopathology by hematoxylin and eosin (HE) staining revealed lung SCC. Immunohistochemical staining confirmed the diagnosis of SCC with positive P40 and CK5/6, negative transcription factor-1 (TTF-1), and napsin A (**Figure 3**). The patient was clinically diagnosed with stage IVA lung SCC, T4N3M1a. Genetic status, including EGFR, ALK, ROS1, KRAS, BRAF, RET, MET, HER2, NRAS, and PI3KA presence, was detected by amplification refractory mutation system (ARMS) with AmoyDx Mutations Detection Kit (Amoy Diagnostics Co., Ltd., Xiamen, China), revealing that the tumor harbored ROS1 rearrangement (**Figure 3**).

The patient was treated with crizotinib (250 mg twice daily) from August 3, 2020. After 3 weeks of treatment, a chest CT scan showed an obvious reduction in tumor size and metastasis (**Figure 1B**), quickly resulting in partial response (PR) according to the Response Evaluation Criteria for Solid Tumors (RECIST, version 1.1) (3). The patient continued receiving crizotinib and developed mild treatment-related adverse events (TRAEs), such as rashes, nausea, and anemia. She stopped molecular targeted therapy for 2 weeks because of severe hypoalbuminemia and secondary bilateral pleural effusion which might be relative to crizotinib (**Figure 1C**). After returning to baseline, crizotinib treatment was reinitiated. The tumor lesions and enlarged LNs further reduced after 4 months (**Figure 1D**). We are still following up with the patient, and she has remained PR for 9 months till the last follow-up (**Figure 1E**).

## Discussion

We report a rare case of a woman diagnosed with lung SCC harboring ROS1 rearrangements, who was extremely sensitive to crizotinib. The ROS1 rearrangements occurred in only 1–2% of patients with non-small cell lung cancer (NSCLC), mainly with adenocarcinoma (2). To date, only six cases of lung SCC with ROS1 have been reported (one was ALK and ROS1 double-rearranged) (1, 2, 4–6). ROS1 rearrangement is very rare in lung SCC (7). Chest CT scans of two previous patients with lung SCC were typical, both presenting a central mass, enlarged mediastinal and hilar LNs (4, 5). However, the CT scan of our patients showed diffuse nodules in the bilateral lung, which we initially suspected as lung adenocarcinoma or lung metastasis from other cancers. We excluded other malignant tumors, mainly by PET–CT. Although we could not completely rule out the possibility of adenosquamous carcinoma, we confirmed the diagnosis of squamous carcinoma based on histopathology of double specimens, immunohistochemistry, and sensitivity of the lesions to crizotinib.

Crizotinib has been approved for the treatment of NSCLC patients with ROS1 rearrangements. As reported in PROFILE 1001 study, crizotinib showed an objective response rate (ORR) of 72%, median progression free survival (PFS) of 19.3 months, median overall survival (OS) of 51.4 months, and 48-months survival probability of 51% in 53 advanced NSCLC patients with ROS1 rearrangements, and TRAEs were mainly grade 1 or 2 per CTCAE v3.0 (8). The efficacy and safety of crizotinib were also confirmed in a phase II OxOnc Study in East Asian patients, with an ORR of 71.7%, median PFS of 15.9 months, and median OS of 32.5 months; most TRAEs were grade 1 or 2 in severity (9). Both two studies showed that the ORR of crizotinib in the treatment of ROS1 rearrangement in NSCLC exceeded 70%. In fact, studies on crizotinib in the treatment of ROS1 rearrangement NSCLC have focused on lung adenocarcinoma since the vast majority of ROS1 mutations occur in the adenocarcinoma subtype. Significantly, the patient in our case was diagnosed with peripheral lung SCC and surprisingly showed a remarkable shrinkage of both tumor lesions and enlarged LNs and has remained PR for 9 months after receiving crizotinib treatment until now. The most commonly reported TRAEs (occurring in more than 25% of patients) of crizotinib in clinical trials were visual disturbances, gastrointestinal toxicities, edema, and elevated ALT/AST, mostly assessed as grade 1/2 in severity (10, 11). Hypoalbuminemia is an uncommon TRAE that was quite severe in this patient. The patient was treated with an albumin infusion. Fortunately, when she resumed crizotinib treatment, severe TRAEs did not recur and she remained PR for a long time. Among the six previous cases, two patients received crizotinib as the first line (5, 6) and two patients received crizotinib as the second line treatment (2, 4). Compared patients with lung SCC receiving first-line treatment with crizotinib, our patient had a better treatment effect and a longer PFS.

## Conclusion

In conclusion, we presented the case of a lung SCC patient with an atypical imaging manifestation and molecular pathology, suggesting that rare ROS1 rearrangement could also unexpectedly occur in patients with lung SCC and is a sensitive target of crizotinib in lung SCC.

## Data Availability Statement

The original contributions presented in the study are included in the article/supplementary material. Further inquiries can be directed to the corresponding author.

## Ethics Statement

Written informed consent was obtained from the individual(s) for the publication of any potentially identifiable images or data included in this article.

## Author Contributions

GY, JW, and YY designed the study and wrote the original draft of the manuscript. JZ, ZY, and QG contributed to the data collections. JY revised the manuscript. WM supervised the study and revised the manuscript. All authors contributed to the article and approved the submitted version.

## Conflict of Interest

The authors declare that the research was conducted in the absence of any commercial or financial relationships that could be construed as a potential conflict of interest.
